# *In vitro* and *in vivo* activities of a trithiolato-diRuthenium complex conjugated with sulfadoxine against the apicomplexan parasite *Toxoplasma gondii*

**DOI:** 10.1016/j.ijpddr.2024.100544

**Published:** 2024-04-27

**Authors:** Ghalia Boubaker, Alice Bernal, Anitha Vigneswaran, Dennis Imhof, Maria Cristina Ferreira de Sousa, Kai Pascal Alexander Hänggeli, Noé Haudenschild, Julien Furrer, Emilia Păunescu, Oksana Desiatkina, Andrew Hemphill

**Affiliations:** a Institute of Parasitology, Department of Infectious Diseases and Pathobiology, Vetsuisse Faculty, University of Bern. Länggass-Strasse 122, 3012, Bern, Switzerland; b Graduate School for Cellular and Biomedical Sciences (GCB), University of Bern, Switzerland; c Department of Chemistry, Biochemistry and Pharmaceutical Sciences, University of Bern, Freiestrasse 3, 3012, Bern, Switzerland

**Keywords:** Organometallic drugs, Sulfadoxine, *Toxoplasma*, Trithiolato diruthenium complex, Proliferation inhibition, Cytotoxicity, Transmission electron microscopy, Splenocytes, *In vivo* efficacy

## Abstract

Organometallic compounds, including Ruthenium complexes, have been widely developed as anti-cancer chemotherapeutics, but have also attracted much interest as potential anti-parasitic drugs. Recently hybrid drugs composed of organometallic Ruthenium moieties that were complexed to different antimicrobial agents were synthesized. One of these compounds, a trithiolato-diRuthenium complex (RU) conjugated to sulfadoxine (SDX), inhibited proliferation of *Toxoplasma gondii* tachyzoites grown in human foreskin fibroblast (HFF) monolayers with an IC_50_ < 150 nM, while SDX and the non-modified RU complex applied either individually or as an equimolar mixture were much less potent. In addition, conjugation of SDX to RU lead to decreased HFF cytotoxicity. RU-SDX did not impair the *in vitro* proliferation of murine splenocytes at concentrations ranging from 0.1 to 0.5 μM but had an impact at 2 μM, and induced zebrafish embryotoxicity at 20 μM, but not at 2 or 0.2 μM. RU-SDX acted parasitostatic but not parasiticidal, and induced transient ultrastructural changes in the mitochondrial matrix of tachyzoites early during treatment. While other compounds that target the mitochondrion such as the uncouplers FCCP and CCCP and another trithiolato-Ruthenium complex conjugated to adenine affected the mitochondrial membrane potential, no such effect was detected for RU-SDX. Evaluation of the *in vivo* efficacy of RU-SDX in a murine *T. gondii* oocyst infection model comprised of non-pregnant outbred CD1 mice showed no effects on the cerebral parasite burden, but reduced parasite load in the eyes and in heart tissue.

## Graphical abstract

**Figure:**
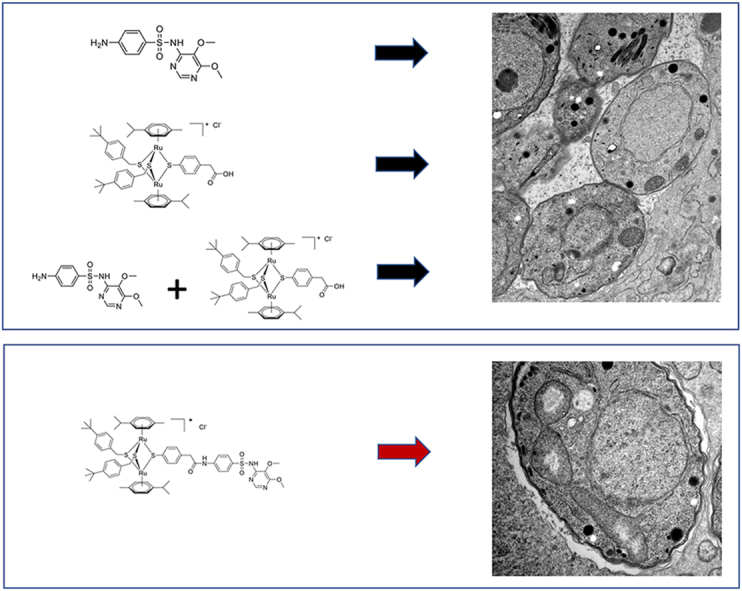

## 1 Introduction

Toxoplasmosis is a zoonotic infectious disease caused by the apicomplexan protozoan *Toxoplasma gondii* (*T. gondii*). Humans acquire infection with *T. gondii* by ingesting tissue cysts in raw/undercooked meat or accidental uptake of oocysts shed with the feces of the feline definitive host from contaminated environments (Attias et al., 2020). Both transmission routes (oocysts and tissue cysts) have nearly equally contributed to global outbreaks of clinical toxoplasmosis (Dubey, 2021; Pinto-Ferreira et al., 2019). Overall, it is assumed that 30% of humanity is infected with *T. gondii* with high inter- and intra-continental variability in prevalence (Flegr et al., 2014). In several intermediate host species including humans, *T. gondii* tachyzoites can be vertically transmitted from mother to the fetus, especially during primary infection, resulting in congenital toxoplasmosis. In immune-competent individuals, 80% of infection cases with *T. gondii* are asymptomatic (Halonen and Weiss, 2013; Luft and Remington, 1992), and most of those patients that exhibit mild symptoms recover within a few weeks without the need for treatment. However, over the past twenty years there has been a significant increase in the number of case reports on severe acquired toxoplasmosis, not only in immune-suppressed but also in immuno-competent individuals (Basit et al., 2018; Carme et al., 2002; De Angelis et al., 2021; Lima et al., 2021; Ramachandran et al., 2014; Sobanski et al., 2013). Furthermore, severe infections were increasingly associated with atypical strains of *T. gondii* as opposed to clonal lineages (Ajzenberg et al., 2002; de-la-Torre et al., 2013). In patients with weakened and compromised immune system such as patients having acquired immune deficiency syndrome (AIDS), *T. gondii* is the most prevalent opportunistic pathogen responsible for central nervous system (CNS) infection cases (Schlüter and Barragan, 2019). For instance, 25% of Ghanaian AIDS patients presenting symptoms of meningitis had cerebral toxoplasmosis (Opintan et al., 2017), and in South India more than 96% of fatal cerebral toxoplasmosis in AIDS patients were associated with atypcial genotypes (Vijaykumar et al., 2018).

The first-line chemotherapeutical treatment of acute toxoplasmosis is comprised of pyrimethamine (PYR), and sulfadiazine (SDZ). Trimethoprim is administered with sulfamethoxazole, and this combination is considered a second-line treatment. Overall, anti-*Toxoplasma* medications can be prescribed frequently during the lifetime as prevention therapy to avoid aggravation of the disease after reinfection or re-activation of tissue-cysts, particularly in high-risk immunocompromised patients (Konstantinovic et al., 2019). Since these drugs interfere with the folate metabolism, a supplementation with folinic acid during treatment is mandatory to reduce severe side effects caused by folic acid deprivation in the patients. Due to its safety profile, the macrolide antibiotic spiramycin is used for treatment of acquired toxoplasmosis in women early during pregnancy to prevent vertical transmission, but established fetal infection cannot be cured. Treatment with the lincosamide clindamycin is a suitable therapeutic alternative, particularly in patients with a contraindication to classical treatment (Rajapakse et al., 2013). None of these drugs pass the blood-brain barrier in sufficient amounts, thus they do not affect the bradyzoites encapsulated in tissue cysts in the CNS. Treatment failure in cerebral toxoplasmosis is considerable since the one-year death rate can be as high as 60%. Furthermore, patients who survive the treatment develop persistent neurological disorders in 37% of cases (Ayoade and Joel Chandranesan, 2023). Drug treatments are frequently associated with adverse side effects. In 2021, a study on adverse outcomes associated with the treatment of *Toxoplasma* infections has designated clindamycin and PYR as most suspicious chemotherapies leading to death of 23 patients among a total of 102 studied cases (Shammaa et al., 2021). Thus, the currently available anti-*T. gondii* chemotherapies are very limited, add to that, none of these drugs can act on the chronic stage. Thus, extensive efforts are being made to develop new chemotherapeutic agents specifically acting on parasitic, but not host cells (Alday and Doggett, 2017).

Platinum-based organometallic compounds have been widely applied as anticancer chemotherapeutics (Gasser et al., 2011). A variety of complexes of other transition metals have been investigated as potential alternatives (Lee et al., 2020). Among those, ruthenium (RU) complexes were shown to exhibit profound anti-parasitic activities (Hm et al., 2017; Munteanu and Uivarosi, 2021), and hundreds of RU-based complexes were screened *in vitro* against *T. gondii* (Basto et al., 2017; Desiatkina et al., 2020, 2021, 2022a, 2022b; Holzer et al., 2023; Păunescu et al., 2021; Studer et al., 2020). However, a major issue with RU-complexes is the *in vitro* toxicity in non-infected host cells, resulting in narrow therapeutic indexes. So far, this has hampered the *in vivo* evaluation of RU-complexes against experimental toxoplasmosis in murine models.

**Fig. 1:**
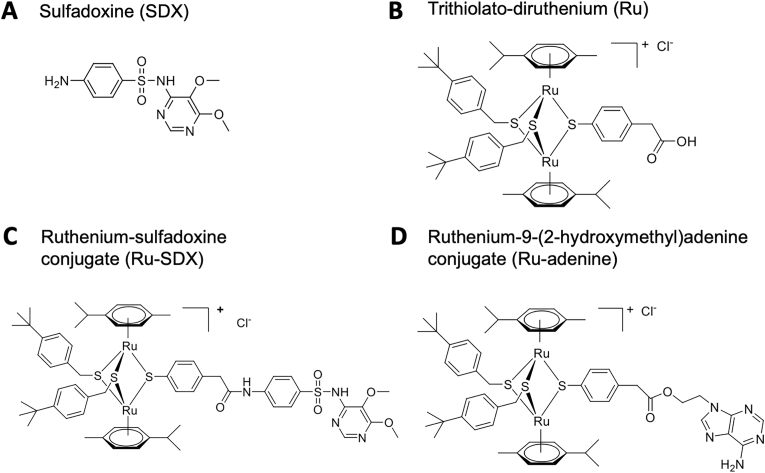
**Chemical stRUctures of compounds used in this study.** The molecular weight of sulfadoxine (SDX) = 310.33; the trithiolato diRuthenium complex (RU) = 1032.19; RU-SDX = 1324.25; RU-9-(2-hydroxyethyl)-adenine = 1193.26.

Recently, hybrid drugs made of organometallic di-ruthenium moieties conjugated to different antimicrobial agents were synthesized (Desiatkina et al., 2021). Interestingly, one of these compounds, RU-SDX conjugate (Fig. 1, C), obtained by conjugating the drug sulfadoxine (SDX; see Fig. 1, A) with a Ru-complex (Fig. 1, B), exhibited decreased host cell toxicity compared to the non-conjugated RU complex alone. The RU-SDX compound inhibited *T. gondii* tachyzoite proliferation *in vitro* very efficiently without interfering in host cell viability, while SDX did not display anti-*Toxoplasma* activity (Desiatkina et al., 2021).

The purpose of this study was to further characterize RU-SDX regarding its efficacy and toxicity profile *in vitro* with respect to *T. gondii*, human foreskin fibroblast (HFF) host cells, and proliferating murine T and B-lymphocytes. Long term drug treatments *in vitro* were carried out to assess whether RU-SDX displays parasiticidal or parasitostatic activity, ultrastructural changes induced by exposure of *T. gondii* to RU-SDX were monitored by TEM, and the potential impact of RU-SDX on the mitochondrial membrane potential (MMP) was investigated. Additionally, safety assessments were carried out to determine whether RU-SDX exposure impacted on early zebrafish embryonic development. Finally, the *in vivo* efficacy of RU-SDX was evaluated in a murine *T. gondii* oocyst infection model comprised of non-pregnant outbred CD1 mice.

## 2 Material and methods

### 2.1 Parasites, host cells and drugs

In this study three *Toxoplasma* strains were used. The transgenic *T. gondii* β-Gal strain derived from *T. gondii* RH (Type I) constitutively expresses β-galactosidase was kindly provided by David Sibley, Washington University, St. Louis, MO, USA, and the *T. gondii* Me49 (a type II strain, kindly provided by Furio Spano, Istituto Superiore di Sanità, Rome) were both maintained by serial passaging in human foreskin fibroblast monolayer cultures in Dulbecco's modified Eagles's medium (DMEM) with 10% fetal calf serum (FCS) and penicillin/streptomycin as previously described (Winzer et al., 2015). Oocysts of the *T. gondii* ShSp1 strain (TgShSp1 (type II) were kindly provided by Complutense University of Madrid (Imhof et al., 2021; Sánchez-Sánchez et al., 2018) and stored at 4 °C until used for experimental infection of CD1 outbred mice.

Compounds used in this study are shown in Fig. 1 and include (A) Sulfadoxine (SDX); (B) the di-ruthenium complex (RU); (C) the hybrid RU-SDX conjugate (RU-SDX); (D) the hybrid RU complex-9-(2-hydroxyethyl)-adenine (RU-adenine). All compounds were synthesized as previously described (Desiatkina et al., 2021; Păunescu et al., 2021). Pyrimethamine (PYR) and the two mitochondrial uncouplers carbonylcyanide-trifluoromethoxyphenylhydrazone (FCCP) and carbonylcyanide-3-chlorophenylhydrazone (CCCP) were purchased from Merck (Darmstadt, Germany). For RU-based drugs and PYR, stock solutions were prepared at 1 mM in dimethylsulfoxide (DMSO) and were stored at −20 °C.

### 2.2 Determination of drug efficacy against *T. gondii*

Effects of drugs on the proliferative capacity of *T. gondii* tachyzoites were investigated using a transgenic *T. gondii* RH strain constitutively expressing β-Galactosidase (*T. gondii* β-Gal (Dc et al., 1997);). Assays were carried out in 96 well plates in the presence or absence of the different compounds. For the quantification of β-Galactosidase activity, a fluorometric assay was applied as previously described (Desiatkina et al., 2021; Păunescu et al., 2021).

To check whether RU-SDX exhibited a static or cidal effect on *T. gondii* cultures, T25 culture flasks were seeded with 3.5 × 10^5^ HFF and maintained for 4 day at 37 °C/5% CO_2_. On day 0, the HFF monolayers were infected with 5 × 10^5^ *T. gondii* Me49 tachyzoites. At 4 h post infection (p.i.) the medium was removed, flasks were washed twice with sterile PBS and fresh medium containing either RU-SDX at 0.25 μM or 0.5 μM, or DMSO (0.05%) as a negative control, were added. Treatments were applied during 3 and 6 days and cultures were maintained in normal DMEM complete media and examined light microscopically daily. After drug removal, flasks were also monitored microscopically daily for one week.

### 2.3 Assessment of toxicity of RU-SDX in HFF, in *Danio rerio* embryos, and in proliferating murine B- and T cells *in vitro*

To determine cytotoxicity of compounds on host cells, HFF were grown to confluency in 96 well plates and were treated with different concentrations of the drugs solubilized in medium during 72 h at 37 °C/5% CO_2_, or were treated with the corresponding concentration of DMSO only (Desiatkina et al., 2020; Holzer et al., 2023; Păunescu et al., 2021). The Alamar blue viability assay was used as previously described (Desiatkina et al., 2020, 2021, 2022a, 2022b; Holzer et al., 2023; Păunescu et al., 2021). In short, resazurin was added to each well at 0.01 mg/ml, and subsequently fluorescence of the produced resorufin was measured at excitation wavelength 544/20 nm and emission wavelength 590/20 nm using a Hidex Sense microplate reader (Hidex, Turku, Finland).

The zebrafish (*Danio rerio*) embryo acute toxicity test was carried out to assess the impact of RU-SDX on early embryo development as previously described (Anghel et al., 2020). RU-SDX was assessed at 0.2, 2 and 20 μM, while the bumped kinase inhibitor BKI-1748 (applied at 50 μM) was used as a positive control resulting in 100% mortality of zebrafish embryos (Anghel et al., 2020). For each test- and control condition, a 24 well plate containing 24 fertilized eggs (1 egg/well) was used. Negative control plates contained 1 ml of E3 medium/osmosis water, whereas eggs in solvent control plates were incubated in E3 medium containing 0.01% DMSO (Anghel et al., 2020). For each treatment, a survival rate was calculated that reflected the degree of either embryo death or malformations during the first 96 h of development in relation to eggs maintained without compound.

To measure the effect of compounds on the proliferative capacity of murine B and T cells stimulated *in vitro* with lipopolysaccharide (LPS) and concanavalin A (ConA), we used the bromodeoxyuridine (BrdU) cell proliferation kit (QIA58, Merck Millipore) as previously described (Păunescu et al., 2021). Spleens were aseptically removed from naïve- or drug-treated mice, then single cell suspensions were prepared (Desiatkina et al., 2020). Splenic lymphocytes were seeded into 96 well-plates and were used either unstimulated (negative control) or stimulated with ConA (5 mg/ml), LPS (10 mg/ml), ConA plus compound or LPS plus compound. The immunosuppressive agent cyclosporine A (CsA) was included as a control (Păunescu et al., 2021; Winzer et al., 2020).

### 2.4 Transmission electron microscopy (TEM)

We examined the cellular ultrastructure of *T. gondii* tachyzoites treated with RU-SDX by TEM as described previously (Anghel et al., 2021; Desiatkina et al., 2020). Briefly, confluent HFF grown in T25 flasks were infected with 10^5^ *T. gondii* Me49 tachyzoites, and infected cultures were maintained for 24 h at 37 °C/5% CO_2._ Subsequently, the medium was removed and medium containing 0.5 μM RU-SDX or 0.05% DMSO (as solvent control) was added, and cultures were maintained at 37 °C/5% CO_2_ during 6, 24 or 48 h. At the different timepoints the samples were fixed and processed for TEM (Anghel et al., 2021; Desiatkina et al., 2020). For this, cultures were washed with 0.1 M sodium cacodylate buffer (pH 7.3) and fixed in 2% glutaraldehyde in cacodylate buffer for 10 min at room temperature (RT). The fixed infected monolayers were gently scraped from the flasks, transferred into Eppendorf tubes, washed with in ice-cold 0.1 M sodium cacodylate buffer and fixed in 2% glutaraldehyde in cacodylate buffer for 2 h at RT. Further preparation steps included post-fixation in 2% osmium tetroxide, pre-staining, stepwise dehydration in ethanol, and embedding in Epon-812 resin. In some instances, T25 flasks containing HFF monolayers infected with *T. gondii* Me49 tachyzoites were treated with FCCP and CCCP for 10 min at 80 and 50 μM, respectively, and samples were prepared for TEM as described above. Following polymerization of the resin at 60 °C overnight, ultrathin (80 nm) sections were cut using an ultramicrotome (Reichert and Jung, Vienna, Austria). Sections were transferred onto formvar-carbon-coated 200 mesh nickel grids (Plano GmbH, Marburg, Germany), stained with Uranyless® and lead citrate (both from Electron Microscopy Sciences, Hatfield PA, USA), and imaging was performed on a FEI Morgagni TEM equipped with a Morada digital camera system (12 Megapixel) operating at 80 kV.

### 2.5 Assessment of interference in the mitochondrial membrane potential (MMP) by the tetramethylrhodamine ethyl ester (TMRE) uptake assay

5 × 10^5^ HFF were seeded into T25 culture flasks in DMEM complete medium and maintained in culture for 72 h to reach ⁓80% confluency. HFF monolayers were left either non-infected or were infected with 2 × 10^5^ *T. gondii* Me49 tachyzoites. After another 48 h, the medium was removed from infected and non-infected T25 flasks and was replaced either by medium containing 0.5 μM RU-SDX, or in the case of non-treated controls, medium containing 0.05% DMSO. As positive controls, the two mitochondrial uncouplers FCCP and CCCP were applied at a concentration of 80 and 50 μM, respectively. PYR was applied at 0.5 μM as a control drug not affecting the MMP, and RU-adenine, used as a conjugate shown earlier to impact on the mitochondrial ultrastructure in *T. gondii* (Anghel et al., 2021; Desiatkina et al., 2022b), was also tested at 0.5 μM (Anghel et al., 2021; Desiatkina et al., 2022b). The infected and non-infected HFF monolayers were treated with RU-SDX, RU-adenine or PYR for 3.5 h, FCCP and CCCP were added during 10 min before the end of the treatment. Cultures receiving no treatment were used as positive control for 100% TMRE uptake. Following the treatments, cells were thrice washed with Hanks' Balanced Salt Solution (HBSS), were incubated with TMRE (500 nM) for 30 min and were subsequently washed five times with HBSS. Finally, 3 ml of HBSS was added to each T25 flask and cells were removed with a cell scraper and were passaged through a G25 needle. The resulting lysates were passed through 3.0-μm polycarbonate membranes and were distributed into 96 well plates (100 μl/well). Fluorescence was measured using a Hidex Sense microplate reader instrument (Agilent Technologies, Santa Clara, CA, USA) with the excitation wavelength 544/20 nm and emitted light collected at 590/20 nm. Value from wells belonging to the same experimental condition were summed and the mean TMRE uptake in each condition was calculated from three biological replicates.

Data comparisons between groups were conducted by student's t-test.

### 2.6 Ethical statement

Protocols involving animals were approved by the Animal Welfare Committee of the Canton of Bern under licenses BE117/2020 and BE48/2023. Animals were handled in strict accordance with practices to minimize suffering. BALB/c and CD1 mice, 6 weeks of age, were purchased from Charles River (Sulzberg, Germany), and were maintained in a common room under controlled temperature and a 14 h/10 h light/dark cycle. Mice were housed in the facility for two weeks for adaptation prior to the experiments and procedures were carried according to the guidelines of the animal welfare legislation of the Swiss Veterinary Office.

### 2.7 Assessment of RU-SDX *in vivo* efficacy in CD1 mice orally infected with *T. gondii* ShSp1 oocysts

Female CD1 mice, 6 weeks of age, were purchased from Charles River (Sulzberg, Germany). They were housed in temperature-controlled animal facilities and were randomly distributed into 4 groups (8 mice/group, with 4 mice per cage), and food and water *ad libitum*. At the age of 8 weeks, mice were experimentally infected by peroral application of 120 TgShSp1 (type II) oocysts (Imhof et al., 2021). Placebo and RU-SDX treatments were started at 3 days p. i., with mice being treated orally by gavage. Group 1 (placebo control) received 100 μl corn oil supplemented with 10% DMSO, while group 2 (treatment group) was administered 5 mg/kg RU-SDX solubilized in 100 μl corn oil/10 % DMSO. These treatments were repeated on days 5, 7, 9 and 11 p. i. During the entire experiment mice were monitored for potential health issues at least twice per day. All mice were sacrificed at 30 days p. i. Brain, eyes, heart, and liver were collected and stored at −20 °C for subsequent DNA extraction and quantification of *T. gondii* load by quantitative PCR (qPCR).

Two additional non-infected groups of 8 mice each were included to study the *in vivo* effect of RU-SDX treatment on spleen cells. One group was treated with RU-SDX as above, the other group was treated with corn oil/10 % DMSO. Following the treatments, mice were euthanized, and the spleens were aseptically removed. Single cell suspensions were prepared, seeded into 96 well plates (Desiatkina et al., 2020), and were used either unstimulated (negative control) or stimulated with ConA (5 mg/ml), LPS (10 mg/ml), ConA plus compound or LPS plus compound. The immunosuppressive agent cyclosporine A (CsA) was included as a control (Păunescu et al., 2021; Winzer et al., 2020). Data comparisons between groups were conducted using a student's t-test.

### 2.8 Determination of parasite load in different tissues by TaqMan-qPCR

Genomic DNA was extracted from the collected samples using the NucleoSpin DNA RapidLyze Kit (Macherey-Nagel, Oensingen, Switzerland) and DNA concentrations were quantified by the QuantiFluor double-stranded DNA (dsDNA) system (Promega, Madison, WI, USA) as previously described (Imhof et al., 2021). DNA concentrations were adjusted to 5 ng/μl, and qPCR reactions were performed in a final volume of 10 μl containing 1x SensiFast master mix (Bioline, Meridian Bioscience), 0.5 μM of reverse and forward primers, 0.1 μM of 529rpeQ-P probe, 0.3 mM dUTP, and one unit of heat-labile Uracil DNA Glycosylase (UDG) (Hänggeli et al., 2022). Amplification was performed in the Bio-Rad CFX 96 QPCR instRUment (Biorad) using the following thermal profile: (1) initial incubation of 10 min at 42 °C, followed by (2) denaturation step of 5 min at 95 °C and (3) 50 cycles of two-step amplification (10 s at 95 °C and 20 s at 62 °C). For quantification, a standard curve was made based on a 10-fold serial dilution of DNA from *T. gondii*, equivalent to tachyzoite numbers ranging from 1 × 10^4^ to 1 per 4 μl. The parasite load was expressed as number of tachyzoites per 20 ng of DNA. Statistical analyses were performed using GraphPad Prism version 5.0 (GraphPad Software, La Jolla, CA, USA). Comparisons of the parasite burdens between groups were conducted with the non-parametric Kruskal-Wallis test, followed by the Mann-Whitney-U test.

## 3 Results

### 3.1 Effects of SDX), RU, a combination of SDX and RU, and the RU-SDX conjugate on *T. gondii* proliferation

Effects of SDX, RU and a combination of equimolar amounts of RU and SDX against *T. gondii* were initially measured at concentrations ranging from 0.1 to 5 μM. Results are summarized in Table 1 showing the percentages of proliferation ± standard deviation in relation to controls without compounds (% of control ± SD). SDX did not impair the proliferation of *T. gondii* tachyzoites at any of these concentrations, whereas RU inflicted a dose-dependent proliferation inhibition already at 1.25 μM, which was more pronounced at 2.5 μM, and proliferation was completely blocked 5 μM. A mixture of SDX and RU combined at equimolar amounts did not improve anti-parasitic activity at 1.25 μM and was even less effective than RU alone at same molarity. However, the hybrid RU-SDX conjugate exhibited a markedly more potent anti-proliferative activity. RU-SDX reduced *T. gondii* proliferation to 77 % already at 0.1 μM, and almost completely abolished tachyzoite proliferation when applied at 1 μM (2% in relation to the non-treated control). At 5 μM, almost no proliferation of *T. gondii* tachyzoites could be measured for RU, the combination RU plus SDX and the hybrid RU-SDX conjugate.

**Table 1:** Effects of SDX, Ru, Ru + SDX and Ru-SDX on the *in vitro* proliferation of *T. gondii* β-gal tachyzoites. Values indicate percentages of proliferation ± standard deviation in relation to controls without compounds (% of control ± SD).

Compound	Conc. (μM)				
	1.1 μM	1 μM	1.25 μM	2.5 μM	5 μM
SDX	b^,^∗111 ± 3	b83 ± 2	∗127 ± 11	∗113 ± 7	∗112 ± 9
Ru	a114 ± 2	a110 ± 2	∗64 ± 10	∗∗∗12 ± 2	∗∗∗0 ± 0
Ru + SDX	102 ± 11	91 ± 9	∗78 ± 6	∗63 ± 7	∗∗∗2 ± 3
Ru-SDX	b^,^*116 ± 1	b^,^∗∗∗11 ± 1	b^,^^a^0 ± 0	∗∗∗0 ± 0	∗∗∗0 ± 0

a reported from (Păunescu et al., 2021) and.

b reported from (Desiatkina et al., 2021).

∗ statistically significant with *P* < 0.05 and.

∗∗∗ statistically highly significant with *P* < 0.001.

**Fig. 2:**
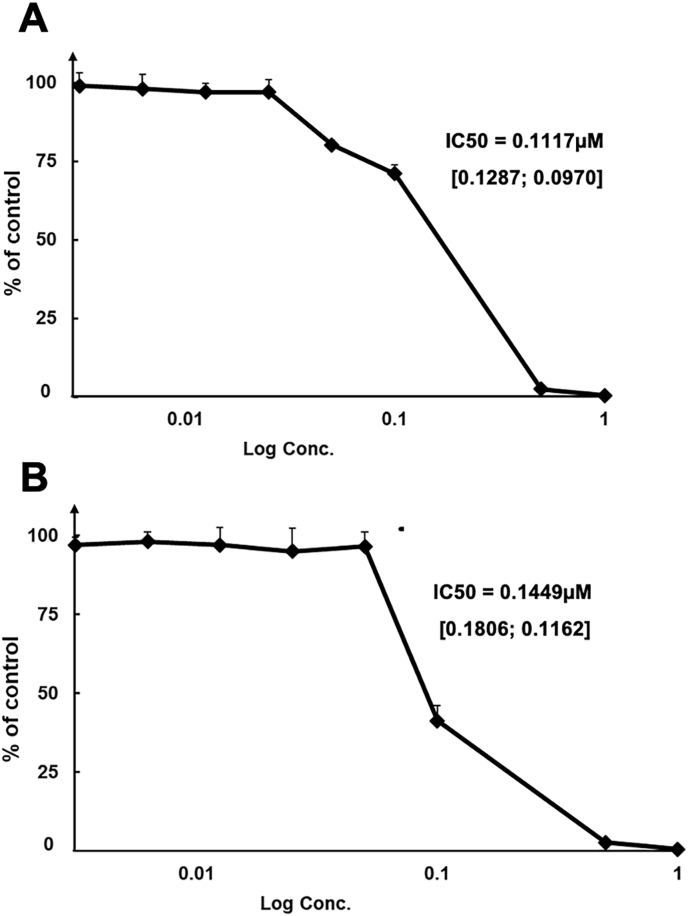
**Dose response curves of *T. gondii* β-Gal tachyzoites exposed to different concentrations of RU-SDX.** (A) shows inhibition obtained when drug treatment was initiated concomitantly to infection, in (B) drug treatment was initiated 3 h post infection. IC_50s_ are given with a 95% confidence interval (CI). Lower and upper limits are shown in square brackets.

In dose-response experiments, RU-SDX was further characterized in terms of IC_50_ values. Similar values were obtained no matter whether drug treatment was applied concomitantly to infection of HFF monolayers or on already infected HFF (IC_50_ = 112 nM and 145 nM respectively) (Fig. 2).

Additional experiments were performed to investigate whether the treatment with RU-SDX was acting parasiticidal or only parasitostatic. For this, continuous treatments during 3 and 6 days were done at a notably high concentration of 0.5 μM, followed by culture of treated specimens in the absence of the drug. For the 6 days treatment, the medium was removed after 3 days and was replaced with fresh medium containing RU-SDX. This showed that after 6 days of continuous treatment with RU-SDX, followed by the removal of the drug and further culture without compound, tachyzoite re-emergence was noted within 4 days. This indicated that RU-SDX did not act parasiticidal *in vitro.*

### 3.2 Toxicity of RU-SDX in HFF host cells

The cytotoxicity of SDX, RU and RU-SDX was assessed in uninfected HFF host cells by employing the Alamar blue assay and determining the percentage of viability ± standard deviation in relation to controls without compounds (% of control ± SD; see Table 2). HFF treated with 2.5, 5 and 10 μM SDX did not display a decrease in viability when compared to untreated cells. In contrast, RU treatment at 10 μM resulted in 100% toxicity, albeit no cytotoxic effects were seen at 2.5 μM and exposure to 5 μM reduced viability of HFF to 70%. RU-SDX treatments also resulted in dose-dependent impairment of HFF viability, with values being slightly lower compared to RU at 2.5 and 5 μM, but at 10 μM viability of HFF was still at 31 %, thus clearly higher than for RU.

**Table 2:** *In vitro* toxicity of SDX, Ru and Ru-SDX to host cells (HFF). Values indicate percentages of viability as determined by Alamar blue assay ± standard deviation in relation to controls without compounds (% of control ± SD).

Compound	Conc. (μM)		
	2.5	5	10
SDX	118 ± 6	106 ± 9	93 ± 5
Ru	100 ± 10	70 ± 10	0 ± 0
Ru-SDX	79 ± 4	64 ± 4	31 ± 9

### 3.3 Impairment of early zebrafish embryo development by RU-SDX

**Fig. 3:**
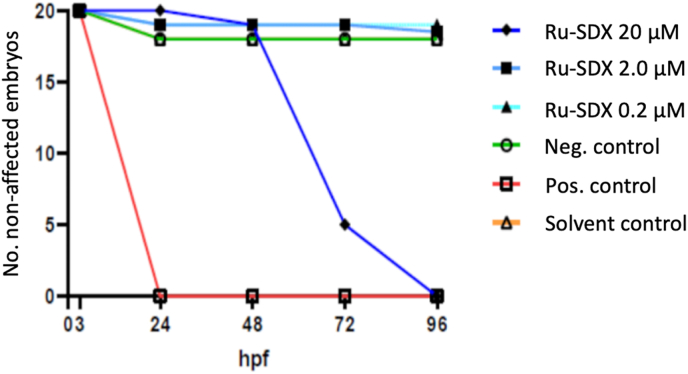
**Survival scores of zebrafish embryos exposed to RU-SDX.** RU-SDX was applied at 0.2, 2 and 20 μM during the first 96 h post-fertilization. The negative control (-Ctrl) remained untreated, the positive control (+Ctrl) was exposed to 50 μM BKI-1748, and the solvent control (Solvent Ctrl) received DMSO only; hpf = hours post-fertilization.

In order to assess whether Ru-SDX treatment could induce teratogenic or embryotoxic effects we assessed the potential impact of RU-SDX treatment on early zebrafish embryo development by exposing freshly collected eggs to either 0.2, 2 or 2 μM of RU-SDX and recording embryo deaths or malformations (Table 3). The survival score (surviving embryos without malformations) was shown to be 95 and 90% upon use of 0.2 and 2 μM RU-SDX (Table 3), but the compound was highly toxic when applied at 20 μM, (no surviving embryos). As displayed in Fig. 3, the peak of mortality of zebrafish embryos treated with 20 μM RU-SDX occurred at 72 h of embryogenesis. In contrast, the positive control drug (BKI-1748 applied at 50 μM) exerted its toxic effect within the first 24 h of treatment.

**Table 3:** Acute toxicity of the hybrid drug Ru-SDX in zebrafish embryos.

	Neg. control E3 medium	Solv. control DMSO 0.1%	Pos. control BKI-1748 50 μM	Ru-SDX 20 μM	Ru-SDX 2 μM	Ru-SDX 0.2 μM
Mortality∗	2/20∗∗∗∗	2/20	20/20	20/20	1/20	1/20
Malformations∗∗	3/20	2/20	0/20	0/20	1/20	0/20
Unaffected embryos∗∗∗	15/20	16/20	0/20	0/20	18/20	19/20

∗ No. of dead embryos.

∗∗ No. embryos with malformations.

∗∗∗ No. embryos with exhibting toxicity.

∗∗∗∗ Total No. of fertilized embryos per assay.

### 3.4 Effects of RU-SDX treatment on *in vitro* proliferation of murine B- and T-cells

**Fig. 4:**
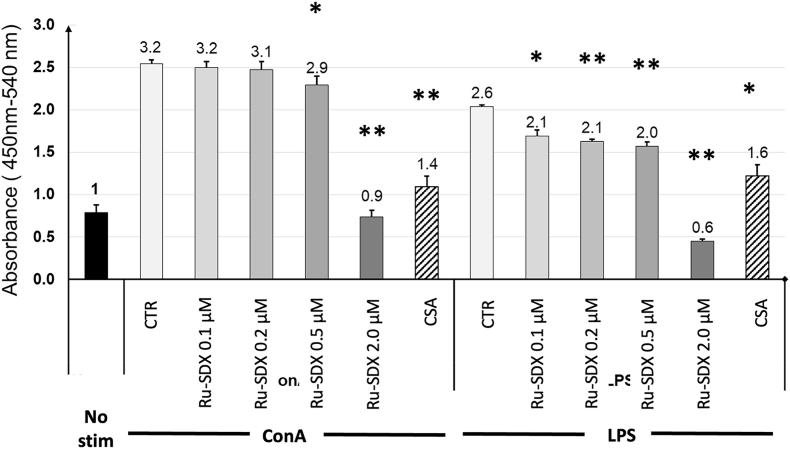
**Dose-dependent effects of RU-SDX on murine T and B cell proliferation *in vitro.*** Spleen cells were prepared from naïve mice and were either left unstimulated (No stim), or were stimulated with ConA or LPS (CTR), or ConA or LPS in the presence of CSA, or ConA or LPS in the presence of RU-SDX at different molarities (0.1–2 μM). All bars represent the mean and standard error of triplicate samples. A value of 1 was set for unstimulated cells, then the stimulation index for each condition is displayed on the top of bars.“. The stars on the top of bars show statistical significance between the between RU-SDX concentrations and CTR-ConA or CTR-LPS: *P < 0.05 and **P < 0.001.

Splenocyte proliferation assays were carried out to evaluate a direct detrimental impact on the immune response by Ru-SDX, such as caused by interfering with activation and proliferation of B- and T-lymphocytes upon antigen recognition. The susceptibility of ConA- and LPS-stimulated native murine spleen cells to RU-SDX treatments *in vitro* was investigated and a potential impairment of T- and B- cell proliferation after ConA and LPS stimulation, respectively, was determined by BrdU-ELISA. A dose-dependent effect on both T- and B-cell proliferation was noted. While no effects were measurable in the presence of 0.1–0.5 μM RU-SDX, the proliferation of stimulated T- and B-cells was significantly inhibited at 2 μM (Fig. 4).

### 3.5 RU-SDX induces transient ultrastructural changes in the mitochondrion of *T. gondii* Me49 tachyzoites

**Fig. 5:**
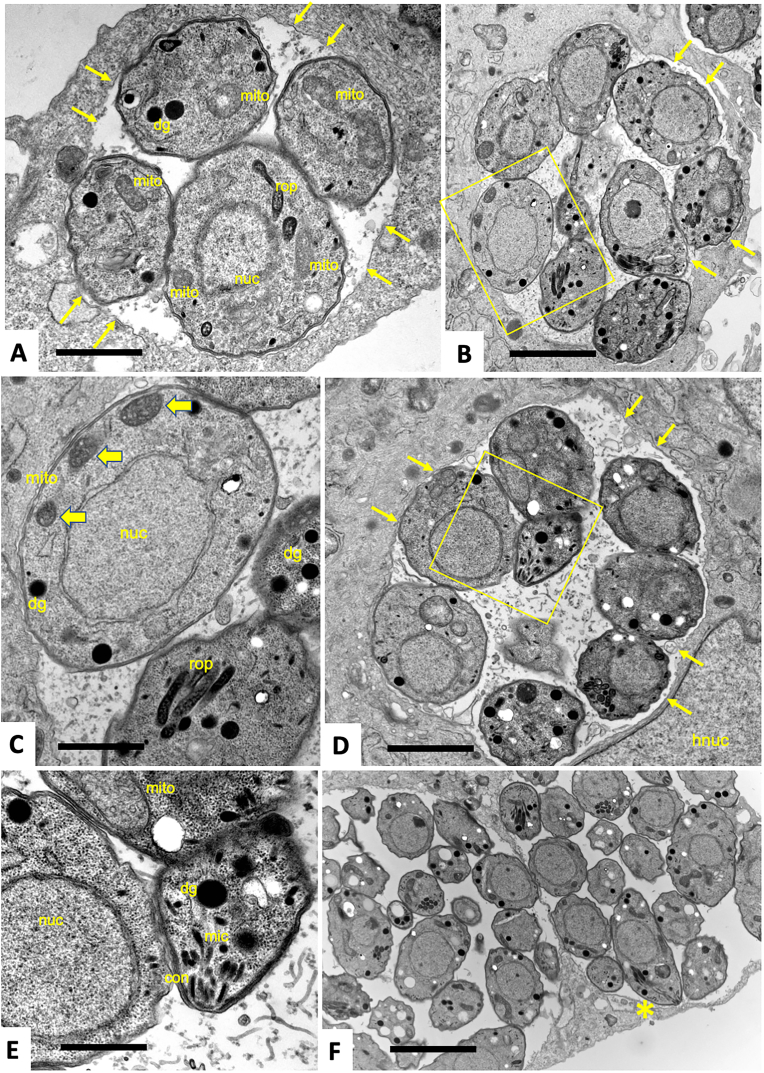
**TEM of *T. gondii* tachyzoites maintained in HFF monolayers in the absence of any drugs (vehicle treatment).** Specimens were fixed and processed after 30 h p. i. (A), 48 h p. i. (B–E) and 72 h p. i. (F). The boxed area in B is shown at higher magnification in C, and the boxed area in D is enlarged in E. Parasites reside in a parasitophorous vacuole enclosed by a parasitophorous vacuole membrane (marked with thin arrows in A, B and D. Secretory organelles such as rhoptries (rop), dense granules (dg) and micronemes (mic), as well as portions of the mitochondrion (mito) are visible. Note the electron dense texture of the mitochondrial matrix in C (marked with thick horizontal arrows). The conoid (con) visible in E marks the apical part of the tachyzoites. The tachyzoites marked with an asterisk in F has a longitudinally oriented section plane; nuc = nucleus, hnuc = host cell nucleus. Bars in A = 0.8 μm; B = 1.2 μm; C = 0.5 μm; D = 1.2 μm; E = 0.4 μm; F = 3 μm.

**Fig. 6:**
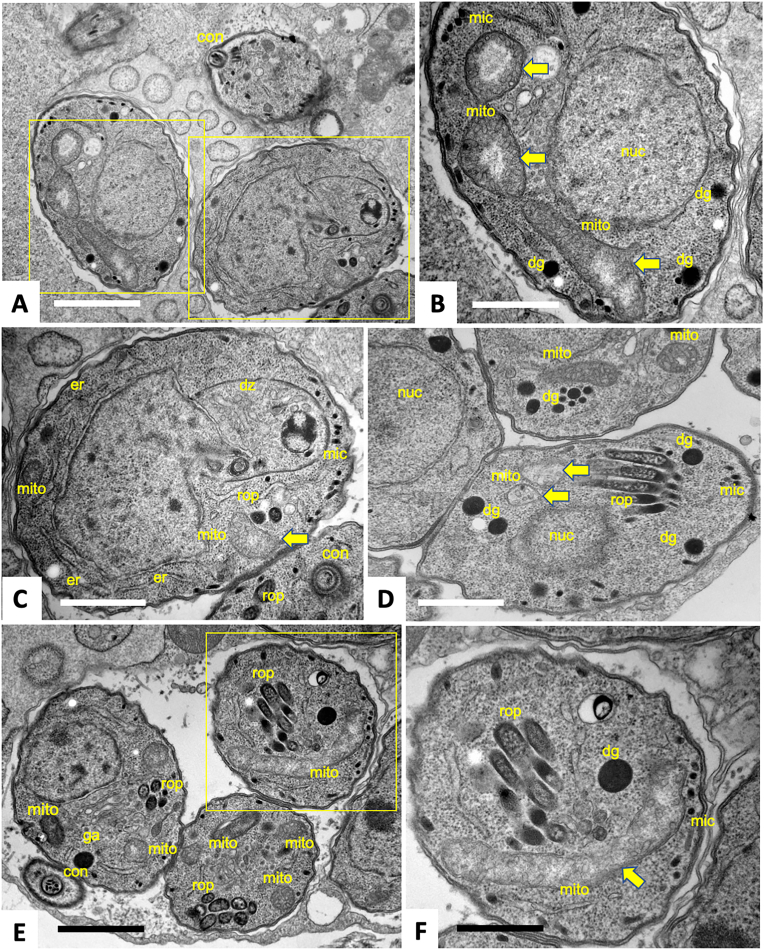
**TEM of *T. gondii* tachyzoites maintained in HFF monolayers in the presence of 0.5 μM RU-SDX.** Specimens were fixed and processed after 6 h (A-C), 24 h (D) and 48 h of treatment (E, F). The two boxed areas in A are enlarged in B and C, the boxed area in E is shown at higher magnification in F. Mitochondria (mito) with a stRUcturally altered and less electron-dense matrix are marked with a thick horizontal arrow; rop = rhoptries, mic = micronemes, dg = dense granules, con = conoid, er = endoplasmatic reticulum, ga = Golgi apparatus, dz = daughter zoite. Bars in A = 1.2 μm; B = 0.6 μm; C and D = 0.8 μm; E = 1.2 μm; F = 0.6 μm.

TEM was used to visualize *T. gondii* Me49 tachyzoites grown in HFF in the presence of the 0.05% DMSO (Fig. 5), and in cultures maintained in the presence of the RU-SDX/0.05% DMSO (Fig. 6). In both, control samples and drug-treated cultures, the intracellular tachyzoites resided in the HFF cytoplasm within a parasitophorous vacuole (PV) surrounded by a parasitophorous vacuole membrane (PVM) (Fig. 5A–D) and underwent proliferation by endodyogeny (visible e. g in Fig. 6 C). However, larger vacuoles containing multiple tachyzoites (as seen in Fig. 6F) were found exclusively in the control cultures maintained in the absence of compound for 48 h.

Tachyzoites have a crescent banana like shape and are 10–15 μm long and 1–2 μm wide; however, by TEM this is not always evident, and the shape depends on the orientation the sections are cut (Ramseier et al., 2021). Typical eukaryotic organelles such nucleus, mitochondrion and Golgi complex are often discernible in the tachyzoite cytoplasm. However, in contrast to mammalian cells, tachyzoites harbor only one mitochondrion, which is composed of branched tubules and exhibits a dense matrix with multiple clearly defined cristae. Only few portions of the mitochondrion are usually visible, again dependent on the section plane (see e.g., Fig. 5, Fig. 6 B).

Other ultrastructural features are exclusively found in apicomplexans, such as a set of secretory organelles named micronemes, rhoptries, and dense granules. Especially micronemes, rhoptries and in some instances, dense granules are integral parts of the apical complex at the anterior part of tachyzoites, also composed of cytoskeletal elements including the conoid at the very anterior tip (see e.g., Figs. 5 E, 6 C, E).

In RU-SDX-treated tachyzoites (Fig. 6), none of these ultrastructural features were visibly altered, except for the mitochondrion. After 6 h of treatment (Fig. 6A–C) the mitochondrial matrix appeared to have lost its characteristic electron dense texture in many areas. After 24 h, tachyzoites that exhibited a similarly altered mitochondrial matrix could be observed, but also other tachyzoites within the same PV displaying intact cristae and an electron dense matrix were visible, like the non-treated controls (Fig. 6 D). A similar picture was evident after 48 h of RU-SDX treatment, although the changes in the mitochondrion were less obvious, and were also not evident in all tachyzoites (Fig. 6 E, F), indicating that mitochondrial impairment could be a transient feature only.

### 3.6 RU-SDX treatment does not impact on the mitochondrial membrane potential (MMP)

**Fig. 7:**
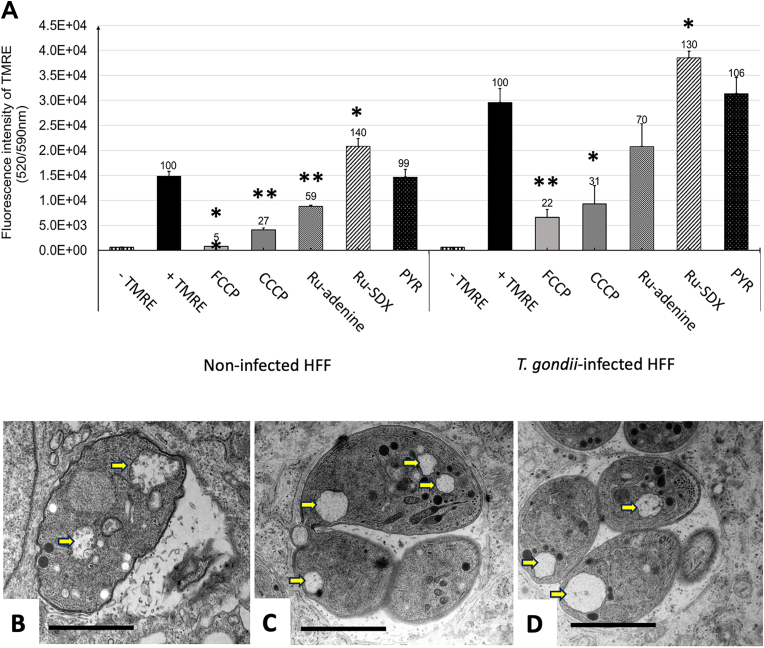
**Impairment of the mitochondrial membrane potential as measured by TMRE uptake and corresponding ultrastructural alterations in *T. gondii* tachyzoites**. A shows TMRE uptake measured in uninfected HFF or *T. gondii*-infected HFF in the presence or absence of either uncouplers (FCCP and CCCP), RU-adenine, RU-SDX or pyrimethamine (PYR). Assays were carried out in T25 tissue culture flasks, and the bars represent the mean of TMRE fluorescence. Standard deviations (SD) are calculated from three biological replicates. 100% of TMRE uptake was set for the control cells in absence of uncouplers or treatments, and the corresponding percentage of TMRE fluorescence intensity is displayed on the top of each bar. Significance analysis was carried out in comparison to the non-treated samples + TMRE (for 100% of TMRE uptake). * = *P* < 0.05; **P < 0.001. Data comparisons between groups were conducted using a student's t-test. B, C and D show TEM micrographs of RU-adenine, FCCP and CCCP treated cultures. Note the altered mitochondria indicated by arrows. Bars in A, B and C = 1.1 μm.

Since RU-SDX induced transient changes in the mitochondrial ultrastructure of *T. gondii* tachyzoites, we investigated whether this compound would possibly have an impact on the MMP. The TMRE-uptake assay was employed in both *T. gondii* infected and non-infected HFF in the presence of absence of RU-SDX. Results displayed in Fig. 7A showed that treatment with RU-SDX for 3.5 h induced an increase in TMRE uptake in uninfected as well as *T. gondii*-infected HFF cultures up to 140 and 130 %, respectively. Treatment of cultures with PYR did not change the TMRE uptake rate (99 and 106 % by HFF or *T. gondii* infected HFF, respectively), while RU-adenine, composed of the same RU-moiety conjugated to 9-(2-hydroxyethyl)-adenine, significantly reduced TMRE accumulation in the mitochondrial matrix, indicating thus a depolarization of the MMP. TEM of RU-adenine treated parasites confirmed that this compound targets the mitochondrion of *T. gondii* tachyzoites (Fig. 7B), which agrees with earlier observations. The two uncouplers CCCP and FCCP, employed here as positive controls, also induced mitochondrial membrane depolarization as indicated by the decrease of TMRE in HFF and *T. gondii* infected HFF (Fig. 7 A). In CCCP and FCCP treated cells, TEM also demonstrated a clear impact on the structural integrity of the mitochondria matrix and cristae (Fig. 7 C, D).

### 3.7 *In vivo* efficacy of RU-SDX treatment in CD1 mice orally infected with *T. gondii* ShSp1 **oocysts**

A preliminary *in vivo* study was conducted in outbred CD1 mice that were experimentally infected with TgShSp1 oocysts. Following oral application of 120 oocysts, a dosage that does not induce severe clinical signs but results in chronic infection, RU-SDX was applied at a dosage of 5 mg/kg/day for 5 days starting at day 3 p. i., with the drug being formulated in corn oil containing 10% DMSO. Higher concentrations of RU-SDX, as well as using other vehicles (PBS ± DMSO, H_2_O ± DMSO, H_2_O + propane-1,2-diol) resulted in precipitation of the compound and were not considered (data not shown).

**Fig. 8:**
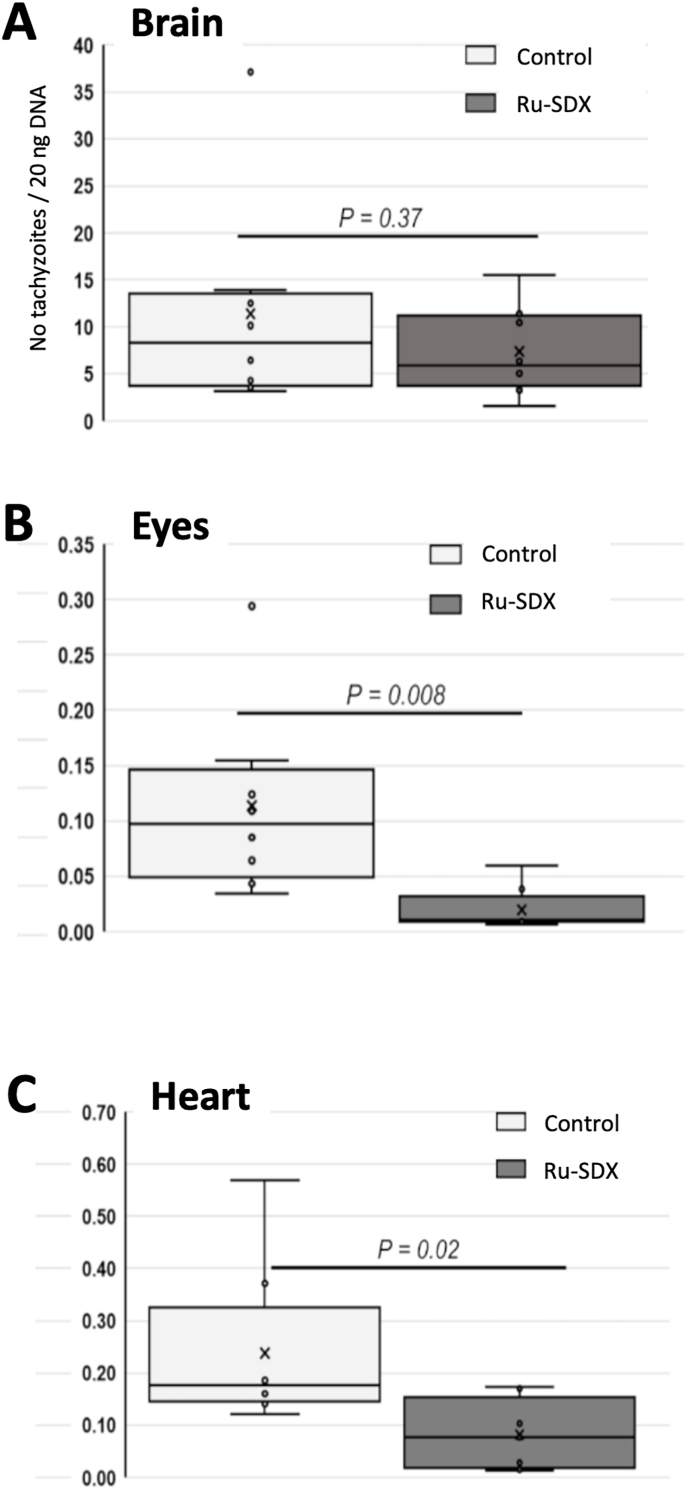
***In vivo* efficacy of RU-SDX in CD1 mice experimentally infected with TgShSp1 oocysts.** Box plots showing the parasite load at 30 days p. i. in placebo control mice (CTR, 0.05% DMSO) and RU-SDX treated mice. The number of parasites in 20 ng DNA extracted from brain (A), eyes (B) and heart (C) was determined using qPCR. Parasite load data are represented as box and whisker (min to max) plots with the x showing the mean per group. Statistics were obtained using Student *t*-test and *P*-values below 0.05 are considered statistically significant.

Neither the infection nor the treatment caused clinical signs, indicating that at this dosage the compound was not toxic. On day 30 p. i., mice were euthanized, the brain tissue, heart and lungs were dissected, and the parasite load was determined by quantitative real time PCR. As can be seen in Fig. 8 A, the treatment with RU-SDX did not result in any reduction of the cerebral parasite load compared to the placebo control group (P = 0.37). Nevertheless, the parasite loads in eyes and in the heart were significantly reduced in the drug treated group (Fig. 8 B, C).

To investigate whether RU-SDX treatment *in vivo* would have an impact on B or T cell proliferation, non-infected mice were similarly treated either with RU-SDX or with vehicle alone, and isolated spleen cells were cultured *in vitro* and stimulated with either ConA or LPS. As seen in suppl, Fig. 1, RU-SDX treatment did not affect the proliferative capacity of these splenocyte populations.

## 4 Discusssion

The currently by far most frequently applied anti-*Toxoplasma* therapeutic agents are SDZ and PYR, which are inhibitors of dihydropteroate synthase (DHPS) and dihydrofolate reductase (DHFR), respectively, both involved the folate synthesis pathway. The development of resistance, as well as an inherent variability in sensitivity, to PYR and SDZ among *T. gondii* strains has been reported, and in the case of SDZ resistance, defined mutations in DHPS were identified (Montazeri et al., 2018). Thus, developing drugs targeting multiple *Toxoplasma* pathways is needed since such therapeutic agents are less prone to drug resistance.

Organometallic ruthenium complexes have shown to exert anti-proliferative effects not only in cancer cells, but also in parasites. While overall it has been suggested that these compounds primarily target DNA (Lin et al., 2018), several studies have shown that they accumulate in the mitochondria and in the endoplasmic reticulum of cancer cells (Basto et al., 2019; Lin et al., 2018). In the light of emerging drug resistance, molecular hybridization has become a promising strategy in drug design, allowing to achieve better selectivity profiles and develop multitarget drug candidates for diseases caused by bacterial infections, but also by parasites such as trypanosomiasis, malaria and toxoplasmosis (Harrison et al., 2018).

Conventional hybrid drug approaches consist of combining biologically active molecules into a compound of higher efficacy and preferably improved specificity (Desiatkina et al., 2021). In our study the two parent drugs SDX and RU exhibited only minor *in vitro* activity against *T. gondii* tachyzoites. SDX is a sulfonamide compound, which is applied in combination with PYR in the treatment or prevention of malaria (brand name Fansidar®). When applied on *T. gondii*, SDX had no effect as shown in this study, and confirming results obtained previously (Mack and McLeod, 1984), but in combination with pyrimethamine SDX inhibited *T. gondii* tachyzoite proliferation more efficiently than PYR alone. When combining SDX with the trithiolato di-ruthenium complex RU used in this study, no improvement of the anti-*Toxoplasma* activity could be seen, but when conjugating SDX to RU, creating the hybrid molecule RU-SDX, a dramatic increase in anti-*T. gondii* efficacy was noted. IC50 values were below 150 nM, no matter whether the drug was added concomitantly to infection or after infection, and cytotoxicity in HFF at 2.5 μM and 5 μM was in the same range as for RU alone, but markedly lower at 10 μM, indicating overall that conjugation RU to SDX has also led to reduced HFF-toxicity.

Thus, the RU-SDX conjugate outperformed the two individual compounds in terms of safety and efficacy, by modifying a known drug through attaching a diRuthenium entity to form a potentially dual-acting drug. Previous studies have investigated very large families of di-Ruthenium compounds and conjugates (Păunescu et al., 2021). While the chemical functionalities that induce good *in vitro* properties, let alone *in vivo*, have not been identified to date, it has been shown that COOH-groups as found in the diruthenium moiety used herein, are detrimental (Păunescu et al., 2021). When considering the Lipinski's rule of five (RO5) for determining the druggability of a new compound, the DiRuthenium moiety hardly checks any boxes: high mass, highly hydrophobic, not very polar, and not able to make more than one hydrogen bond. Sulfadoxine, on the other hand, is a drug that mostly conforms to the RO5. Considering the DiRU-SDX conjugate, the mass is higher, but its polarizability is much higher, and it has a good number of hydrogen acceptors and donors. It can therefore be speculated, following the RO5, that DiRU-SDX has increased the activity and selectivity of SDX, as well as maintained drug-like physicochemical properties.

While HFF monolayers represent cells with a low metabolic turnover, splenocytes that are stimulated with ConA or LPS, represent actively multiplying cells. No effects were seen at concentrations up to 0.5 μM, but a clear impairment of proliferation was noted when splenocytes were exposed to 2 μM of RU-SDX, indicating that toxicity in actively proliferating cells is increased. Another safety assessment was a zebrafish embryo acute toxicity test, which informed on a potential teratogenic effect of RU-SDX. RU-SDX was shown not to interfere in early embryo development when fertilized eggs were exposed to 0.2 and 2 μM RU-SDX, while at 20 μM, death of all embryos was noted. 20 μM is a concentration unlikely to be achieved *in vivo*; thus, the overall toxicity values for this compound are promising. Low to high toxicity of ruthenium-based metallodrugs in the zebrafish model has been previously documented (Karas et al., 2021; Mello-Andrade et al., 2018; Yusoh et al., 2023).

Continuous *in vitro* treatment of *T. gondii* infected HFF with RU-SDX at 0.5 μM for 6 days prevented tachyzoite proliferation initially, but did not eliminate the parasites, leading to regrowth shortly after drug removal. This is not surprising, since it has been shown earlier that it is intrinsically difficult to completely eliminate *T. gondii* tachyzoites by *in vitro* drug treatments, demonstrated previously for many other compounds including decoquinate- and artemisinin-derivatives (Ramseier et al., 2021), bumped kinase inhibitors (Imhof et al., 2021), pentamidine derivatives (Kropf et al., 2012), as well as other ruthenium-based organometallic drugs (Barna et al., 2013).

While RU-SDX does admittedly not act parasiticidal, its parasitostatic activity allowed to investigate the ultrastructural changes that occurred during treatment *in vitro*. Drug-induced changes were most evident at 6 h p. i., and concerned only the mitochondrial matrix, cristae and associated structures, while other organelles in *T. gondii* tachyzoites remained unaffected. These alterations were not dramatic, and a loss of the electron dense matrix texture was seen only in some sections of the mitochondrion, while other portions remained seemingly intact, and the overall size of the mitochondrion was not notably affected. These rather subtle changes were partially lost after 24 and 48 h, indicating that mitochondrial impairment was transient only, and the parasite population started to recover.

While our findings suggest that the primary target of RU-SDX is the mitochondrion, earlier reports utilizing TEM described ruthenium-based drugs such as RU-adenine (Anghel et al., 2021) or a RU-coumarin hybrid (Desiatkina et al., 2020) to cause mitochondrial swelling, with the organelle occupying a larger part of the cytoplasm compared to non-treated cells. In addition, RU-adenine induced similar effects in *Trypanosoma brucei* (Anghel et al., 2021). For *T. gondii* and *T. brucei*, RU-adenine affinity chromatography coupled to mass spectrometry revealed that mitochondrial proteins were the by far most significant RU-adenine binding partners (Anghel et al., 2021), and the mitochondrion is readily targeted by ruthenium compounds also in other cell types, such as A2780 cancer cells (Basto et al., 2019).

We applied TMRE assay to investigate whether targeting of the mitochondrion could have an impact on the mitochondrial membrane potential (MMP), similar to what has been previously found for *T. brucei* bloodstream forms treated with dinuclear thiolato-bridged arene ruthenium complexes (Jelk et al., 2019). TMRE incorporates into mitochondria that exhibit an intact MMP. FCCP and CCCP are classical protonophores, and in *Toxoplasma* and other protozoa such as *Plasmodium* FCCP has been used as uncoupler (Scarpelli et al., 2021; Seidi et al., 2018), but specific effects on the mitochondria ultrastructure have not been demonstrated by TEM. We show here that the use of FCCP and CCCP lead to reduced TMRE dye uptake, and this can be correlated with mitochondrial swelling and loss of matrix and cristae as shown by TEM. RU-adenine treatment exerted a similar depolarizing effect, resulting in diminished TMRE signal (Perry et al., 2011) and similar effects on mitochondrial structures. While modest depolarization of the MMP is known to attenuate mitochondrial ROS generation (Aklima et al., 2021; Vyssokikh et al., 2020), long-lasting and complete mitochondrial depolarization results in activation of mitophagy where mitochondria are recycled without inducing cell death (Jin et al., 2010; Picca et al., 2023). In *Toxoplasma*, a study showed that treatment with ionophore monensin induces mitochondrial damage by decreasing MMP; this in turn forces *Toxoplasma* to undergo cell cycle arrest and thereafter an autophagy-like cell death (Charvat and Arrizabalaga, 2016).

Surprisingly, RU-SDX exerted the opposite effect. The increased TMRE uptake in RU-SDX treated non-infected and *T. gondii* infected HFF (plus 30–40% in relation to non-treated controls) points towards presence of a hyperpolarized, rather than a depolarized, mitochondrial membranes in parasites as well as in host cells, resulting from a decline in mitochondrial proton concentration and increased dye uptake compared to a control (Perry et al., 2011). In immune cells, hyperpolarized mitochondria are a source of reactive oxygen species (ROS) production (Mills et al., 2016; Siska et al., 2017), and hyperpolarization of the mitochondrial inner membrane has been described as an early event associated with mitochondria-dependent apoptosis in Caco-2 intestinal cells (Giovannini et al., 2002; Matsuyama and Reed, 2000). Possibly these differences in MMP alterations induced by RU-SDX and RU-adenine are reflected in the differential mitochondrial alterations seen by TEM.

The potential therapeutic value of RU-SDX was investigated in a *T. gondii* oocyst infection model using non-pregnant CD1 mice infected with TgShSp1 oocysts, which were treated with RU-SDX formulated in corn oil/DMSO at 5 mg/kg/day for 5 consecutive days. The treatment did not lead to a decreased cerebral parasite load, but a decreased parasite load in the heart and in the eyes. To date, pharmacokinetic and pharmacodynamic properties of RU based drugs, particularly the trithiolato-bridged dinuclear Ruthenium (II)-arene compounds, are still completely unknown, which in turn hinders an accurate estimation of the optimal dose for maximal efficacy (al Jalali and Zeitlinger, 2020). In addition, *in vivo* metabolism is a main issue challenging the efficacy of hybrid drugs (Zhao et al., 2022). Besides, the fact that treatment of mice with RU-SDX significantly reduced parasitemia in heart and eyes but not in the brain, suggests that this compound cannot pass the blood brain barrier. We speculate that this is due to its high molecular weight, which is five times higher than PYR. It has been shown that passive diffusion through tight junctions is only possible for lipophilic drugs at a molecular weight lower than 400–600 g/mol (Wu et al., 2023), while the molecular weight amounts up to RU-SDX is over 1300 g/mol. In addition, a previously carried out biodistribution analysis of a RU-analogue by inductively coupled plasma mass spectrometry in BALB/c mice indicated that ruthenium was not detected in the brain (Basto et al., 2019).

*In vivo* drug interactions, particularly with immune cells, can contribute to therapy failure as it has been shown for an antimicrobial peptide, AMP6027, in the treatment of murine toxoplasmosis (Müller et al., 2022). However, in this case, it is unlikely that immune mediated effects account for the lack of *in vivo* efficacy, since T- and B- cells from RU-SDX-treated did not show any impaired proliferation rate compared to non-treated mice. Therefore, occurrence of a compromised/weakened immunity RU-SDX treated mice as a therapy related adverse event can be excluded. Although *in vitro* proliferation assays using splenocytes from naïve mice, showed that RU-SDX inhibits proliferation T- and B- lymphocytes at 2 μM, but not at 0.5 μM. This concentration is high and unlikely to be achieved *in vivo.*

In conclusion, we demonstrated that by conjugating two biologically inactive molecules into one it was possible to generate a hybrid with promising and selective anti-*T. gondii* activity and low host cell toxicity. Summarizing, *in vitro* functional assays RU-SDX suggest that the primary drug target for trithiolato-bridged dinuclear Ruthenium (II)-arene compounds is the mitochondrion, and depending on the conjugation partner they cause either depolarization of hyperpolarization of the MMP. While a preliminary *in vivo* experiment resulted in a treatment failure in the murine model, this highlights the importance of pharmacokinetics and pharmacodynamics studies to be carried out in future studies towards an improved optimization of *in vivo* trials. In addition, different formulations of RU-SDX and/or derivatives should be evaluated, including lipid-based nanocarriers or nanosuspensions, micelles, nanoparticles and solid dispersions.

## Funding

This work was funded by the 10.13039/100000001Swiss National Science Foundation projects 310030_184662 and CRSII5_173718. AB was supported through the Magistère Genetique of the University of Paris.

## CRediT authorship contribution statement

**Ghalia Boubaker:** Conceptualization, Formal analysis, Methodology, Supervision, Writing – original draft, Writing – review & editing. **Alice Bernal:** Investigation, Methodology. **Anitha Vigneswaran:** Investigation, Methodology. **Dennis Imhof:** Investigation, Methodology, Supervision. **Maria Cristina Ferreira de Sousa:** Investigation, Methodology. **Kai Pascal Alexander Hänggeli:** Investigation, Methodology, Supervision. **Noé Haudenschild:** Investigation, Methodology. **Julien Furrer:** Funding acquisition, Investigation, Methodology, Resources, Writing – review & editing. **Emilia Păunescu:** Investigation, Resources, Writing – review & editing. **Oksana Desiatkina:** Investigation, Resources, Writing – review & editing. **Andrew Hemphill:** Conceptualization, Data curation, Funding acquisition, Project administration, Visualization, Writing – review & editing.

## Declaration of competing interest

None, no conflict of interest.

## Figures and Tables

**Suppl Fig. 1:**
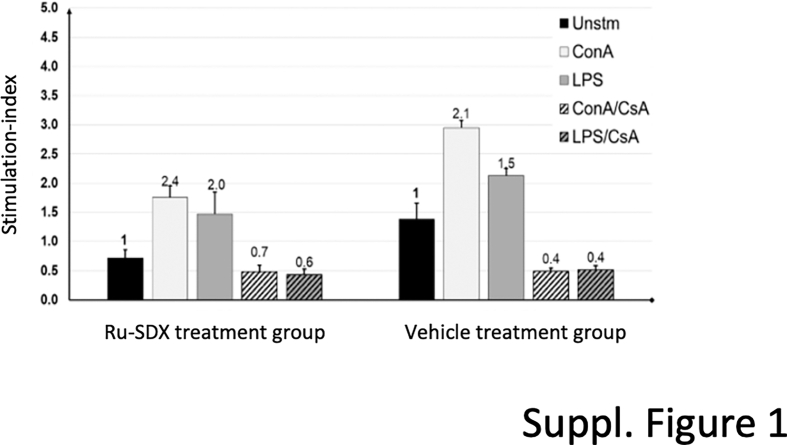
**RU-SDX *in vivo* treatment does not affect the proliferative capacities of murine B- and T-cells stimulated with ConA and LPS, respectively.** Spleen cells were prepared from RU-SDX treated mice and from vehicle treated mice, and were left, and were left either unstimulated (Unstm), or were stimulated with ConA or LPS, or ConA or LPS in the presence of CSA. All bars represent the mean and standard error of triplicate samples. A value of 1 was set for unstimulated cells, then the stimulation index for each condition is displayed on the top of bars.
